# Supplementary material for: Design and Synthesis of C-1 Methoxycarbonyl Derivative of Narciclasine and Its Biological Activity
Source: Molecules. 2022 Jun 14;27(12):3809. doi: 10.3390/molecules27123809 (PMC9230822; doi:10.3390/molecules27123809)
Supplement: Supplementary file 1 [file molecules-27-03809-s001.zip › molecules-1770659-supplementary.pdf]

Design and synthesis of C-1 derivatives of narciclasine and their biological activities

Lihi Habaz,*[a] Korey Bedard,[a] Mitchell Smith,[b] Liqin Du,[b] Alexander Kornienko,*[b] Tomas Hudlicky[a]

[a]Department of Chemistry, Brock University, 1812 Sir Isaac Brock Way, St. Catharines, ON L2*S* 3A1, Canada
lhabaz@brocku.ca

[b]Department of Chemistry and Biochemistry, Texas State University, San Marcos, TX 78666, *USA*
a_k76@txstate.edu

Spectral data provided for all novel compounds.

TABLE OF CONTENTS

Compound **21**……………………………………………………………………..2

Compound **22**……………………………………………………………………..3

Compound **23**……………………………………………………………………..4

Compound **24**……………………………………………………………………..5

Compound **27**……………………………………………………………………..6

Compound **28**……………………………………………………………………..7

Compound **29**……………………………………………………………………..8

Compound **30**……………………………………………………………………..9

Compound **10**………………………………………………………………………10

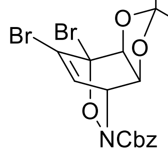
Br
Br
O
O
O
NCbz

21

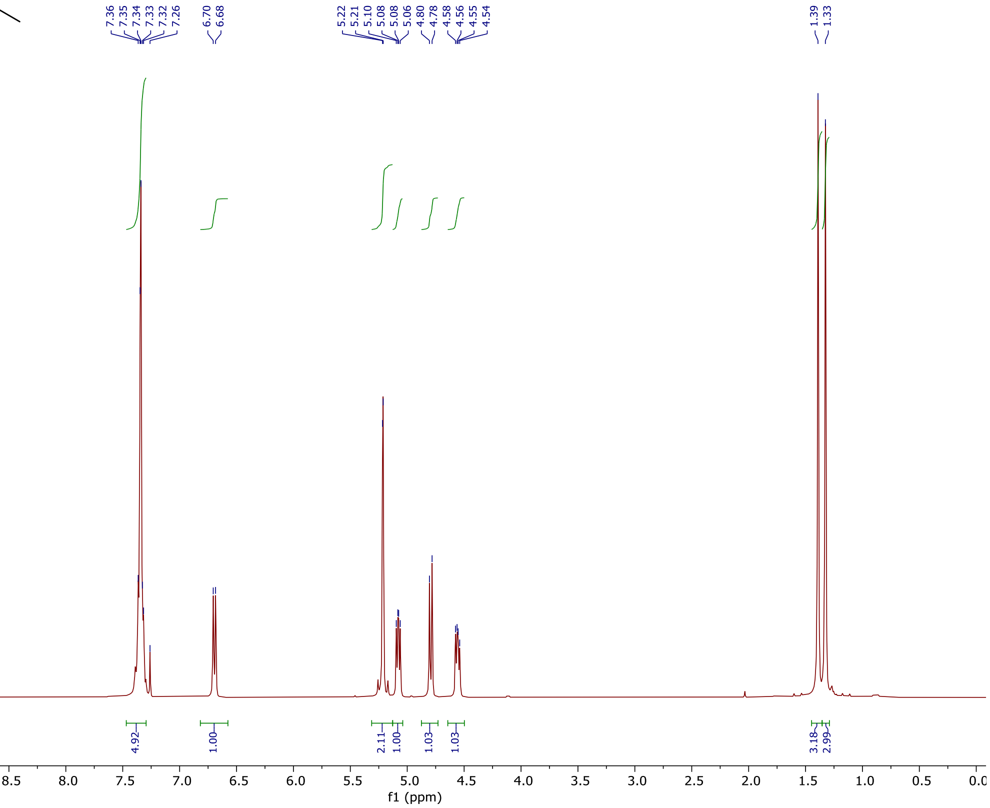
7.36
7.35
7.34
7.33
7.32
7.26
6.70
6.68
5.22
5.21
5.10
5.08
5.08
5.06
4.80
4.78
4.58
4.56
4.55
4.54
1.39
1.33
4.92
1.00
2.11
1.00
1.03
1.03
3.18
2.99
f1 (ppm)

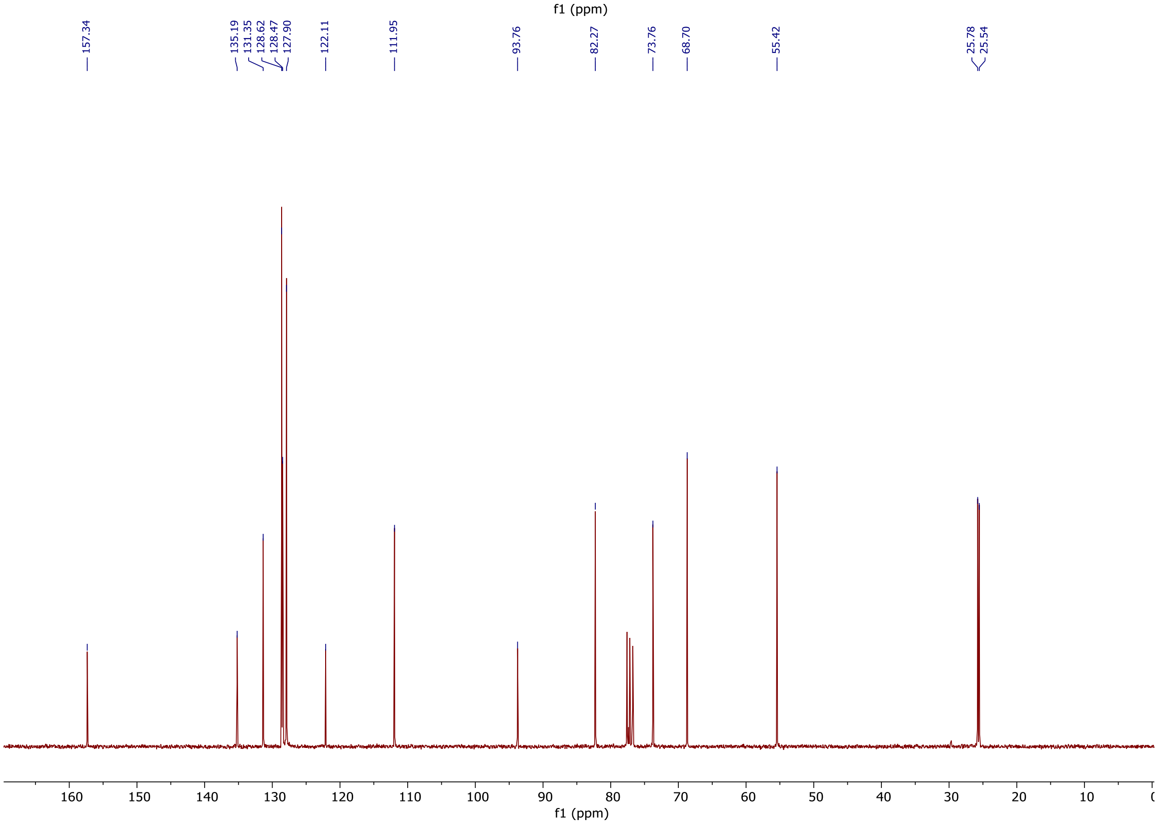
157.34
135.19
131.35
128.62
128.47
127.90
122.11
111.95
93.76
82.27
73.76
68.70
55.42
25.78
25.54
f1 (ppm)

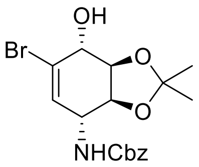
OH
Br
O
O
NHCbz

22

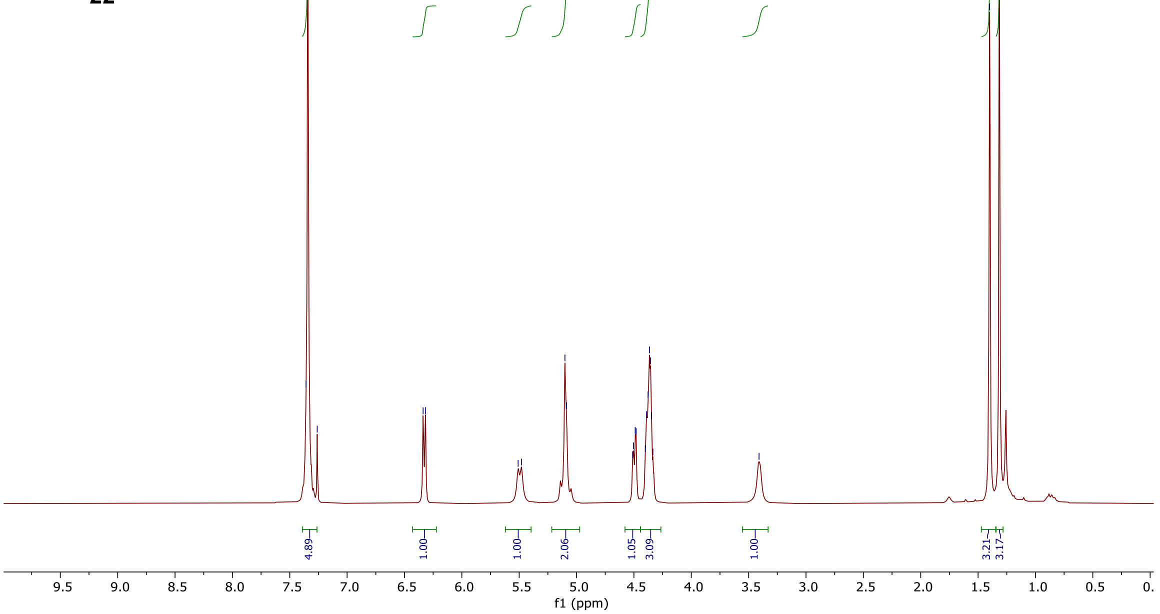
7.36
7.34
7.26
6.34
6.32
5.51
5.48
5.10
5.09
4.51
4.50
4.49
4.48
4.40
4.39
4.38
4.36
4.35
4.35
4.33
3.41
1.40
1.31
4.89
1.00
1.00
2.06
1.05
3.09
1.00
3.21
3.17
9.5 9.0 8.5 8.0 7.5 7.0 6.5 6.0 5.5 5.0 4.5 4.0 3.5 3.0 2.5 2.0 1.5 1.0 0.5 0.
f1 (ppm)

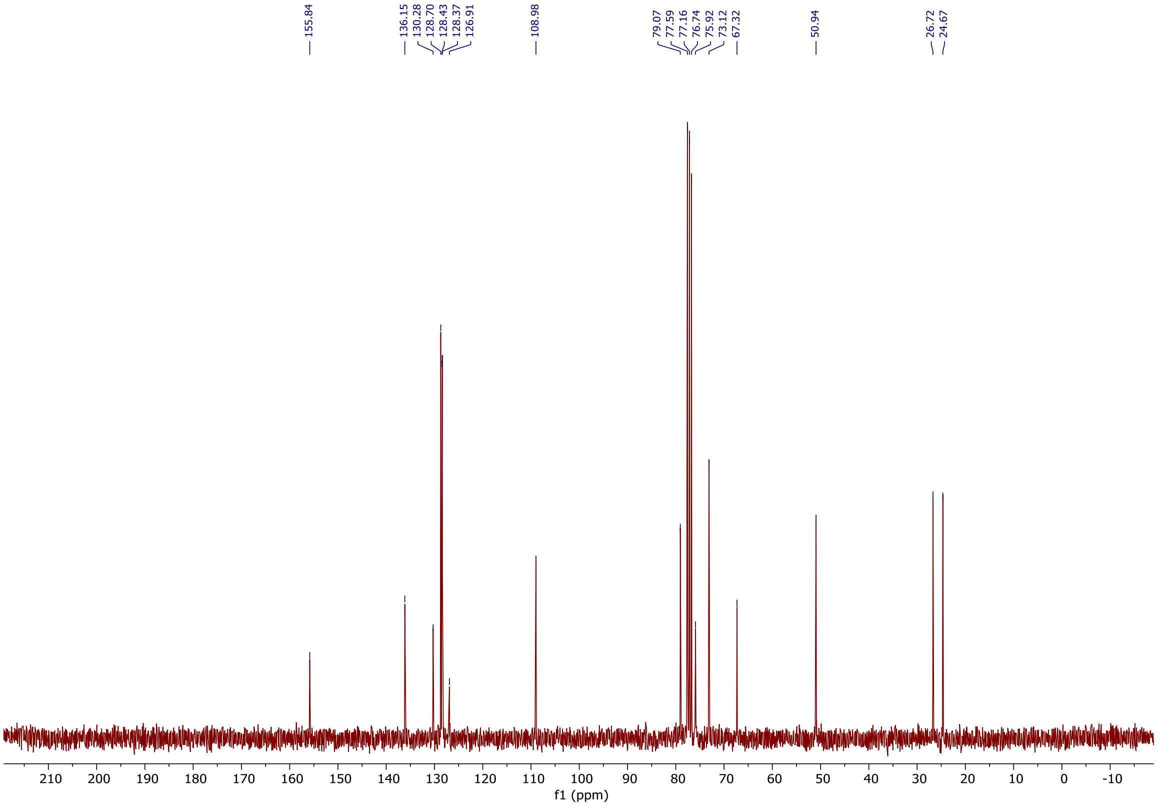
155.84
136.15
130.28
128.70
128.43
128.37
126.91
108.98
79.07
77.59
77.16
76.74
75.92
73.12
67.32
50.94
26.72
24.67
210 200 190 180 170 160 150 140 130 120 110 100 90 80 70 60 50 40 30 20 10 0 -10
f1 (ppm)

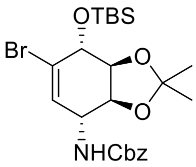
OTBS
Br
O
O
NHCbz

23

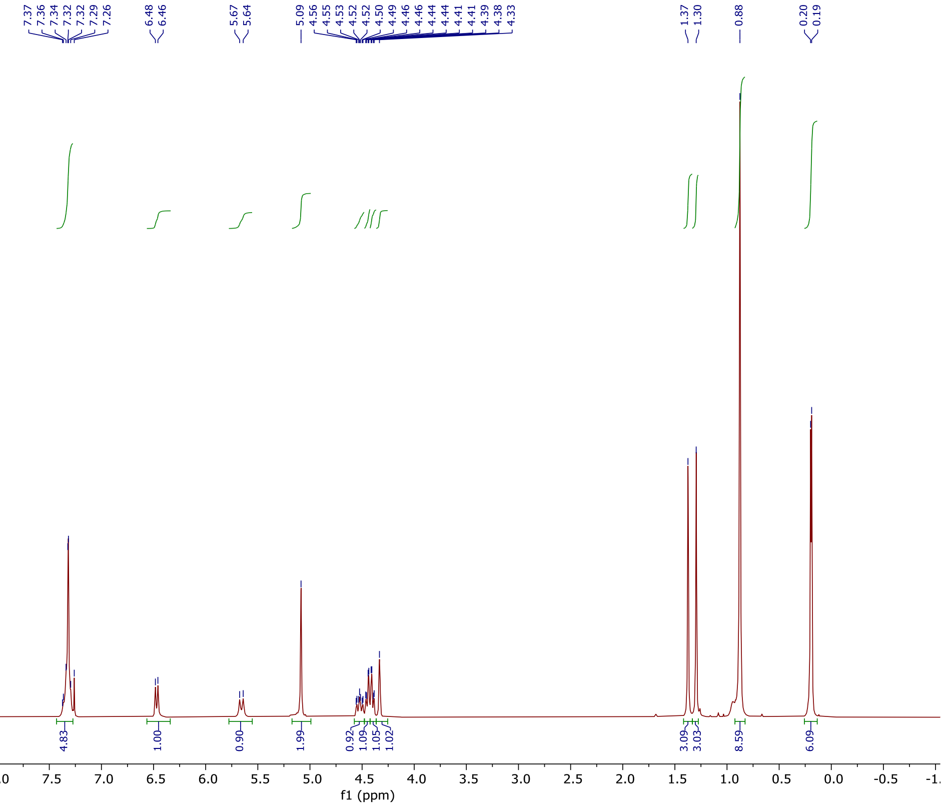
7.37 7.36 7.34 7.32 7.32 7.29 7.26
6.48 6.46
5.67 5.64
5.09 4.56 4.55 4.53 4.52 4.52 4.50 4.49 4.46 4.44 4.44 4.41 4.41 4.39 4.38 4.33
1.37 1.30
0.88
0.20 0.19
4.83
1.00
0.90
1.99
0.92 1.09 1.05 1.02
3.09 3.03
8.59
6.09
f1 (ppm)

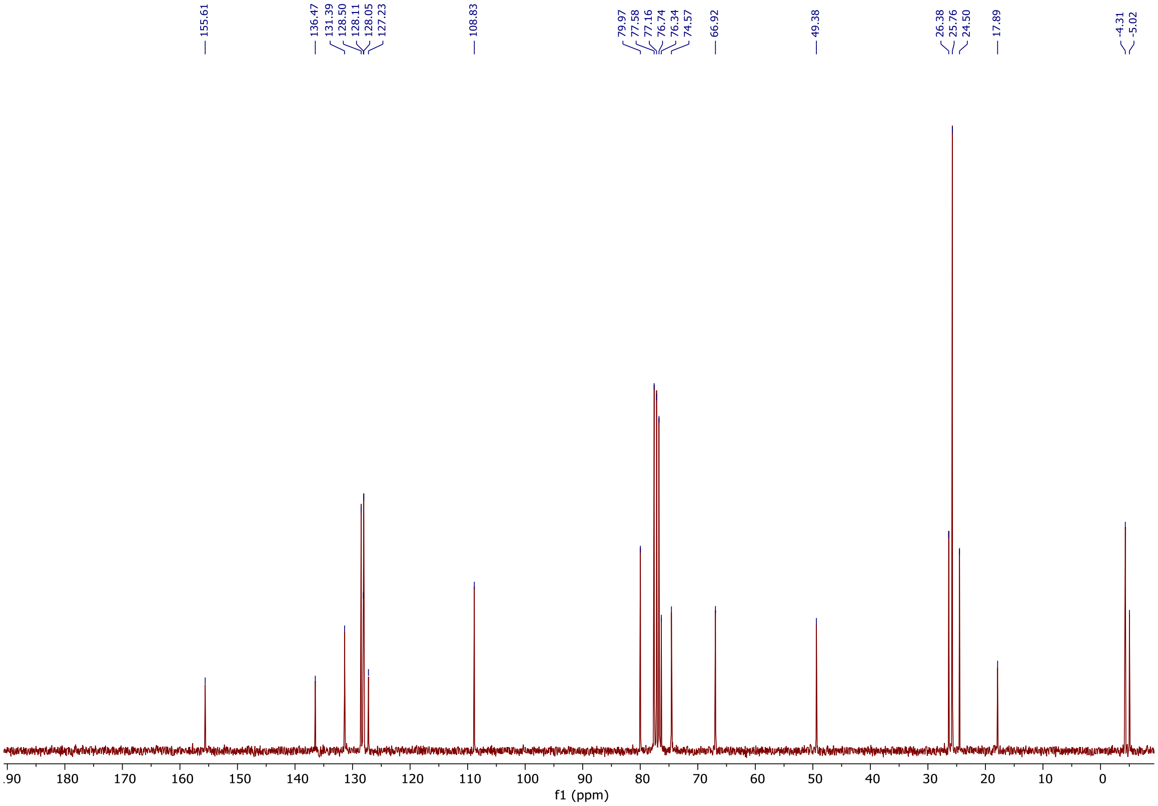
155.61
136.47 131.39 128.50 128.11 128.05 127.23
108.83
79.97 77.58 77.16 76.74 76.34 74.57
66.92
49.38
26.38 25.76 24.50
17.89
-4.31 -5.02
f1 (ppm)

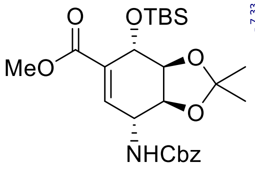
OTBS
MeO
O
NHCbz

24

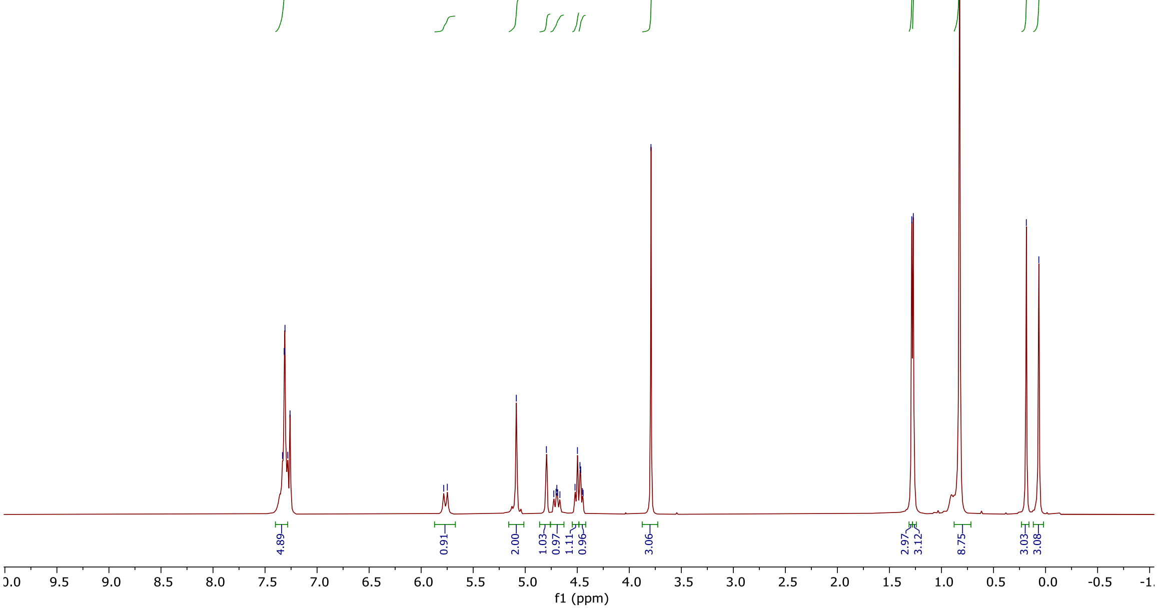
7.33
7.32
7.31
7.28
7.26
5.78
5.75
5.09
4.80
4.73
4.70
4.70
4.69
4.67
4.52
4.50
4.47
4.47
4.45
4.44
3.79
1.29
1.27
0.83
0.18
0.06
4.89
0.91
2.00
1.03
0.97
1.11
0.96
3.06
2.97
3.12
8.75
3.03
3.08
f1 (ppm)

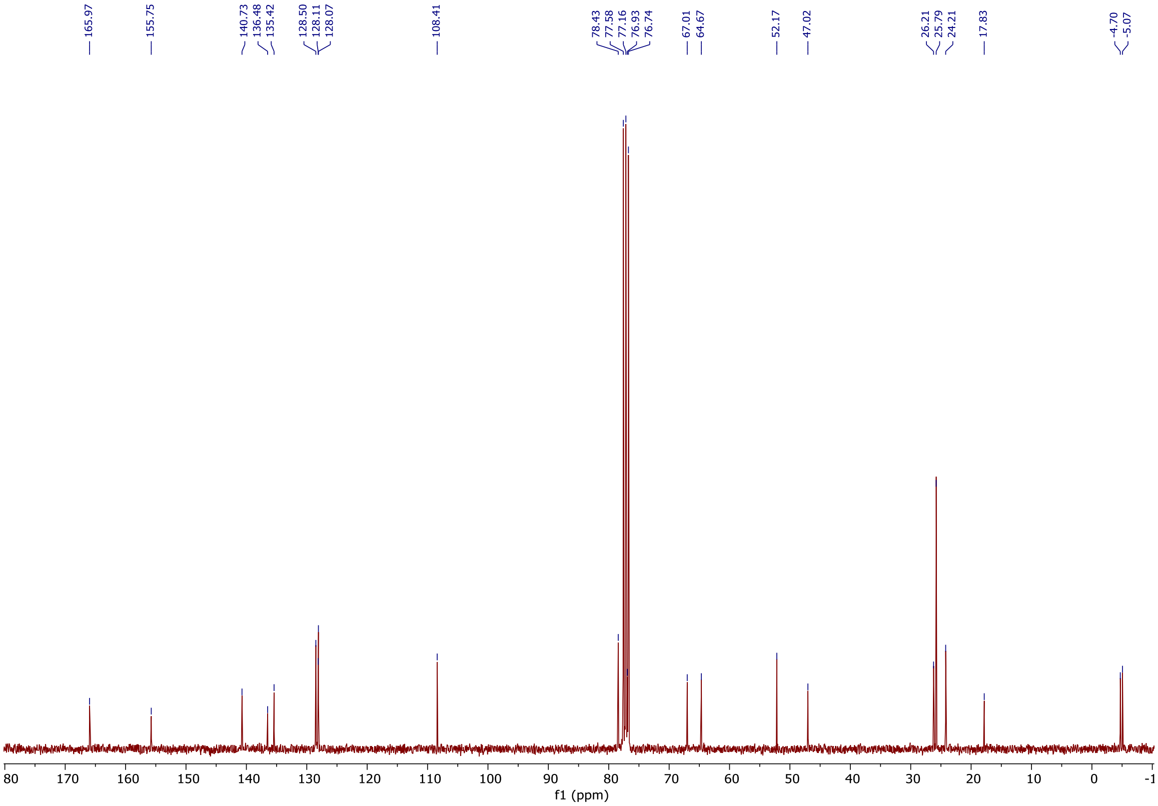
165.97
155.75
140.73
136.48
135.42
128.50
128.11
128.07
108.41
78.43
77.58
77.16
76.93
76.74
67.01
64.67
52.17
47.02
26.21
25.79
24.21
17.83
-4.70
-5.07
f1 (ppm)

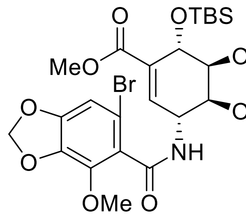
O
OTBS
MeO
Br
O
O
NH
O
O
OMe
O

27

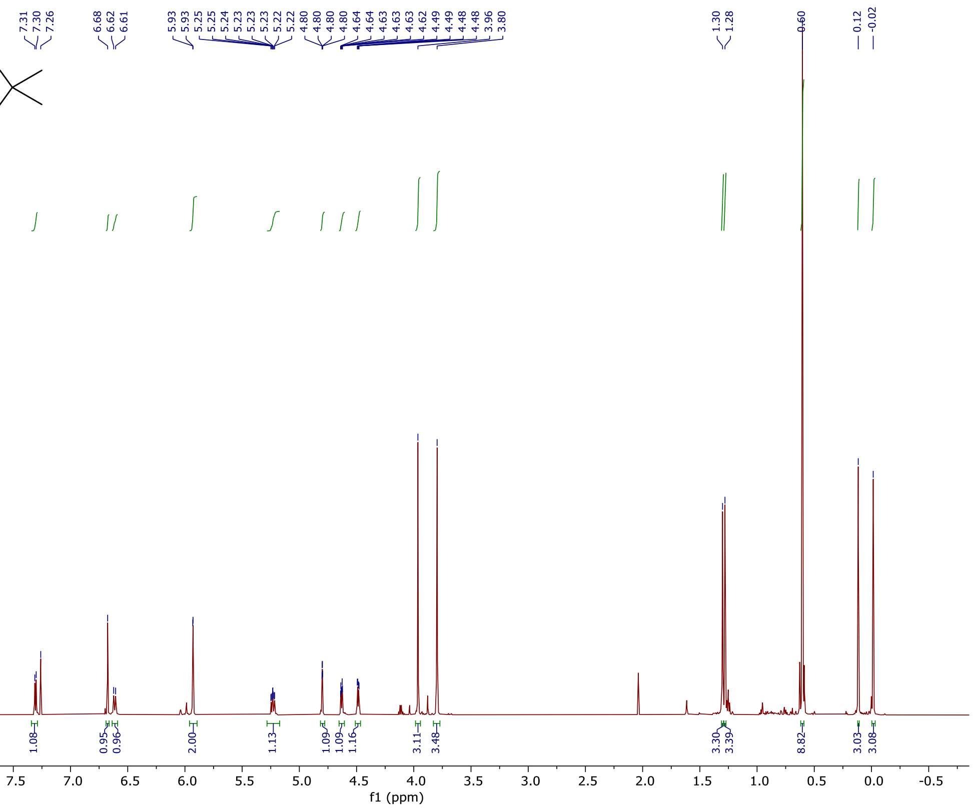
7.31
7.30
7.26
6.68
6.62
6.61
5.93
5.93
5.25
5.25
5.24
5.23
5.23
5.23
5.22
5.22
4.80
4.80
4.80
4.80
4.64
4.64
4.63
4.63
4.62
4.49
4.49
4.48
4.48
3.96
3.80
1.30
1.28
0.60
0.12
-0.02
1.08
0.95
0.96
2.00
1.13
1.09
1.09
1.16
3.11
3.48
3.30
3.39
8.82
3.03
3.08
9.0 8.5 8.0 7.5 7.0 6.5 6.0 5.5 5.0 4.5 4.0 3.5 3.0 2.5 2.0 1.5 1.0 0.5 0.0 -0.5
f1 (ppm)

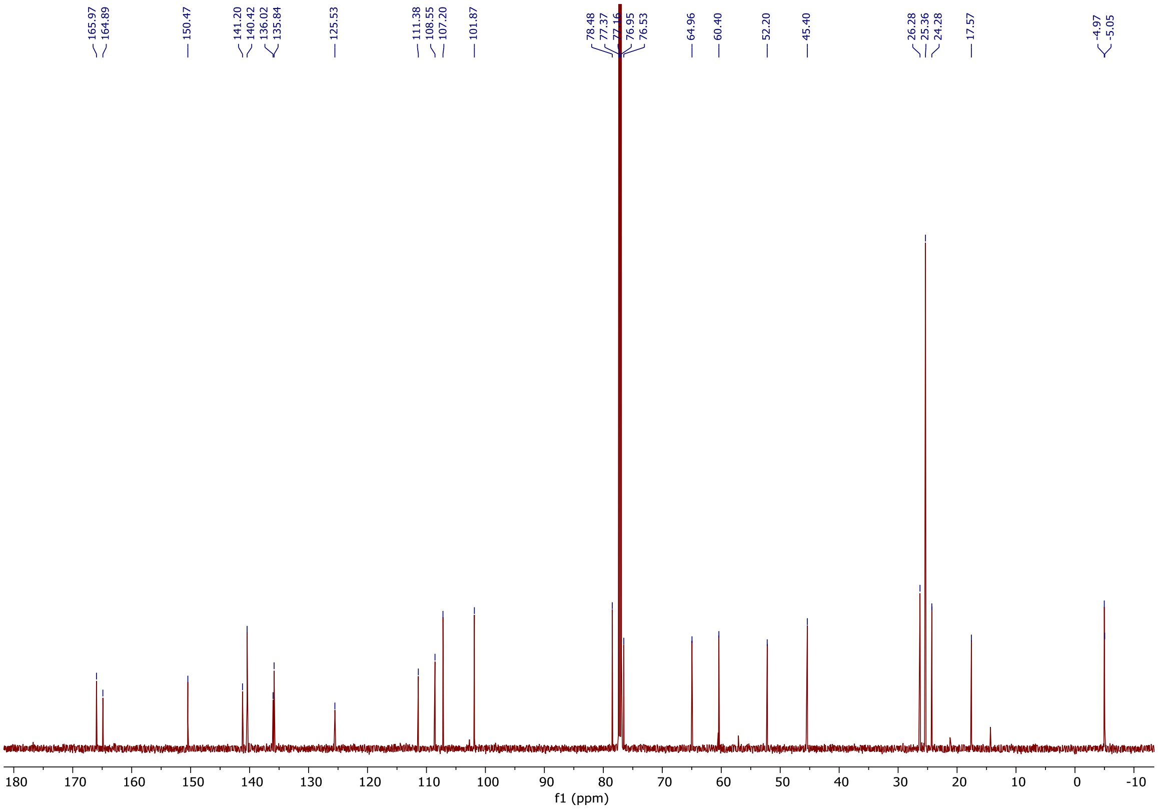
165.97
164.89
150.47
141.20
140.42
136.02
135.84
125.53
111.38
108.55
107.20
101.87
78.48
77.37
77.16
76.95
76.53
64.96
60.40
52.20
45.40
26.28
25.36
24.28
17.57
-4.97
-5.05
180 170 160 150 140 130 120 110 100 90 80 70 60 50 40 30 20 10 0 -10
f1 (ppm)

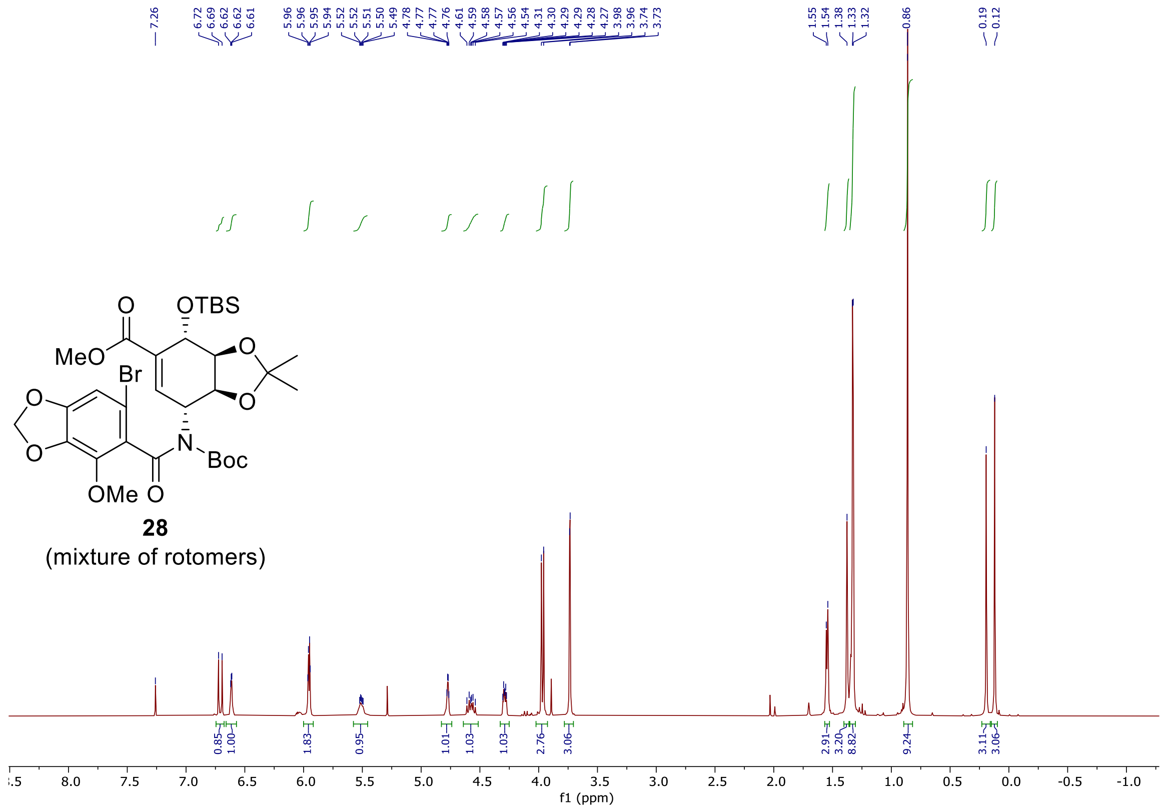

28

(mixture of rotomers)

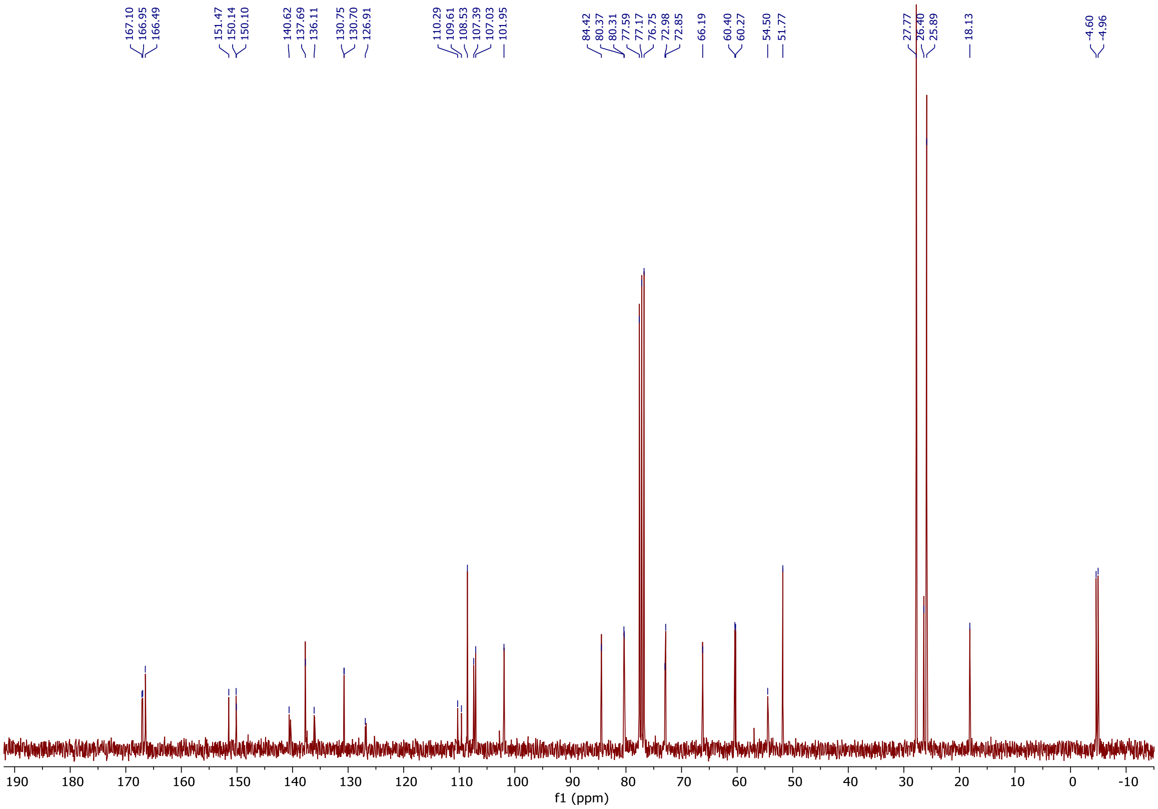

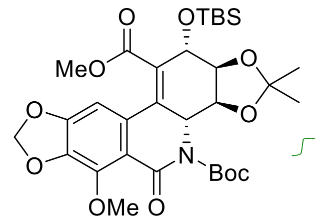
OTBS
MeO
O
N
Boc
OMe

29

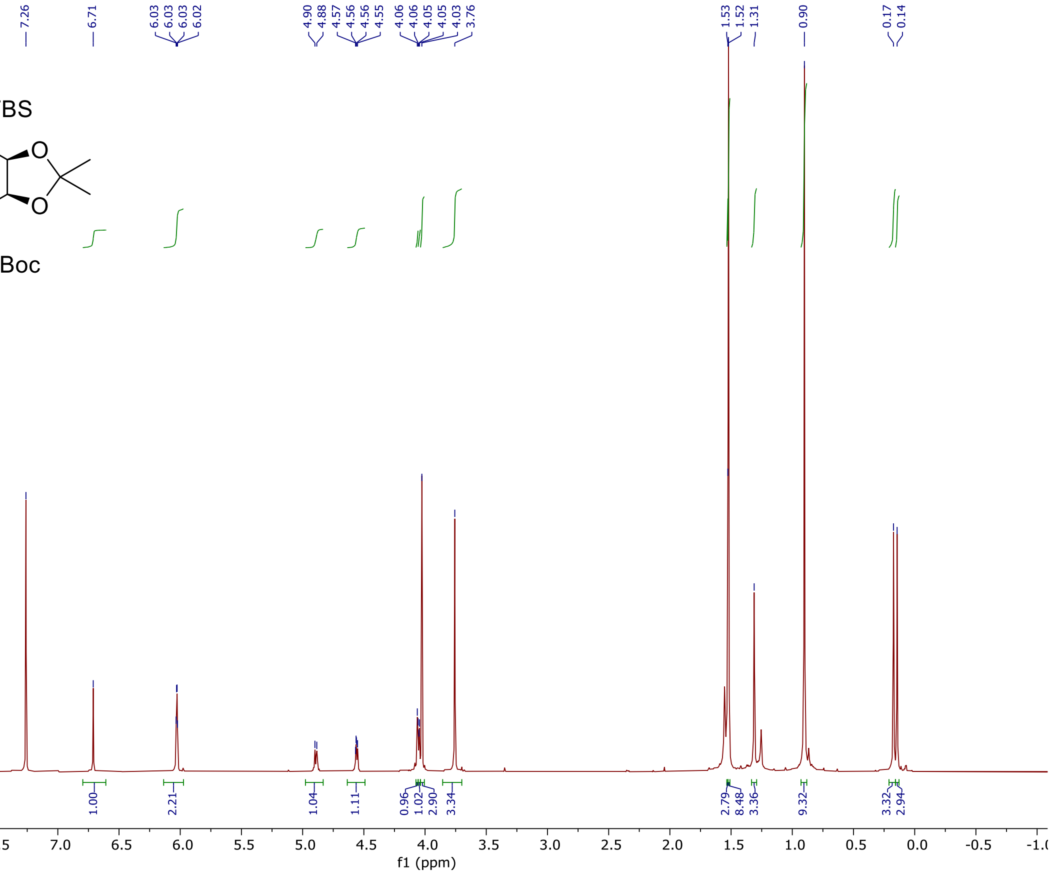
7.26
6.71
6.03
6.03
6.03
6.02
4.90
4.88
4.57
4.56
4.56
4.55
4.06
4.06
4.05
4.05
4.03
3.76
1.53
1.52
1.31
0.90
0.17
0.14
1.00
2.21
1.04
1.11
0.96
1.02
2.90
3.34
2.79
8.48
3.36
9.32
3.32
2.94
f1 (ppm)

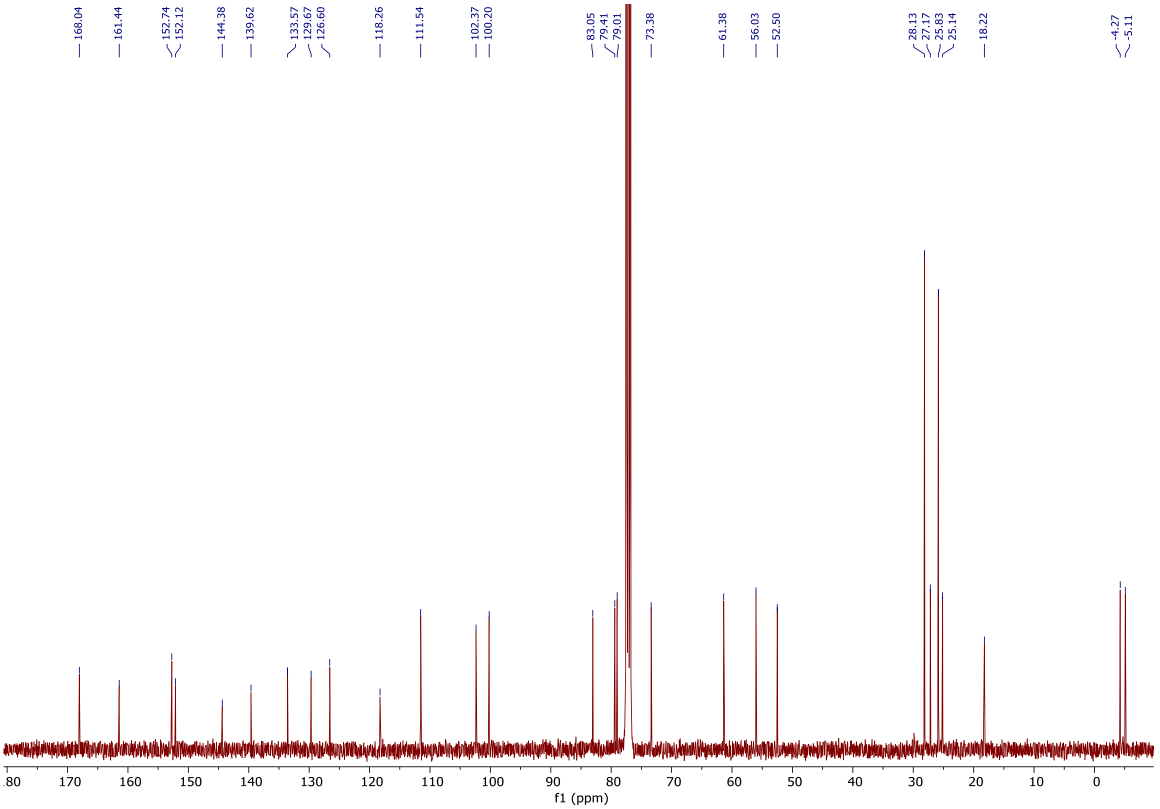
168.04
161.44
152.74
152.12
144.38
139.62
133.57
129.67
126.60
118.26
111.54
102.37
100.20
83.05
79.41
79.01
73.38
61.38
56.03
52.50
28.13
27.17
25.83
25.14
18.22
-4.27
-5.11
f1 (ppm)

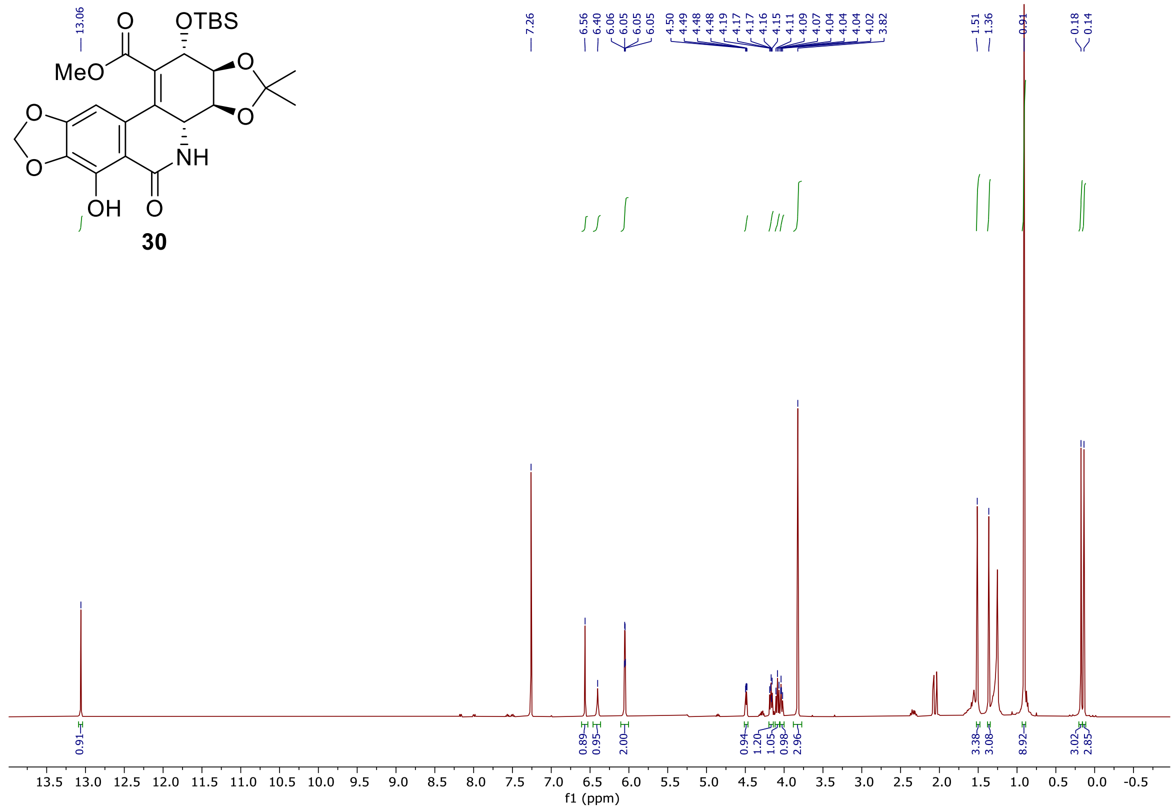

OTBS
MeO
NH
OH
30
13.06
7.26
6.56
6.40
6.06
6.05
6.05
6.05
4.50
4.49
4.48
4.48
4.19
4.17
4.17
4.16
4.15
4.11
4.09
4.07
4.04
4.04
4.04
4.02
3.82
1.51
1.36
0.91
0.18
0.14
0.91
0.89
0.95
2.00
0.94
1.20
1.05
0.98
2.96
3.38
3.08
8.92
3.02
2.85
f1 (ppm)

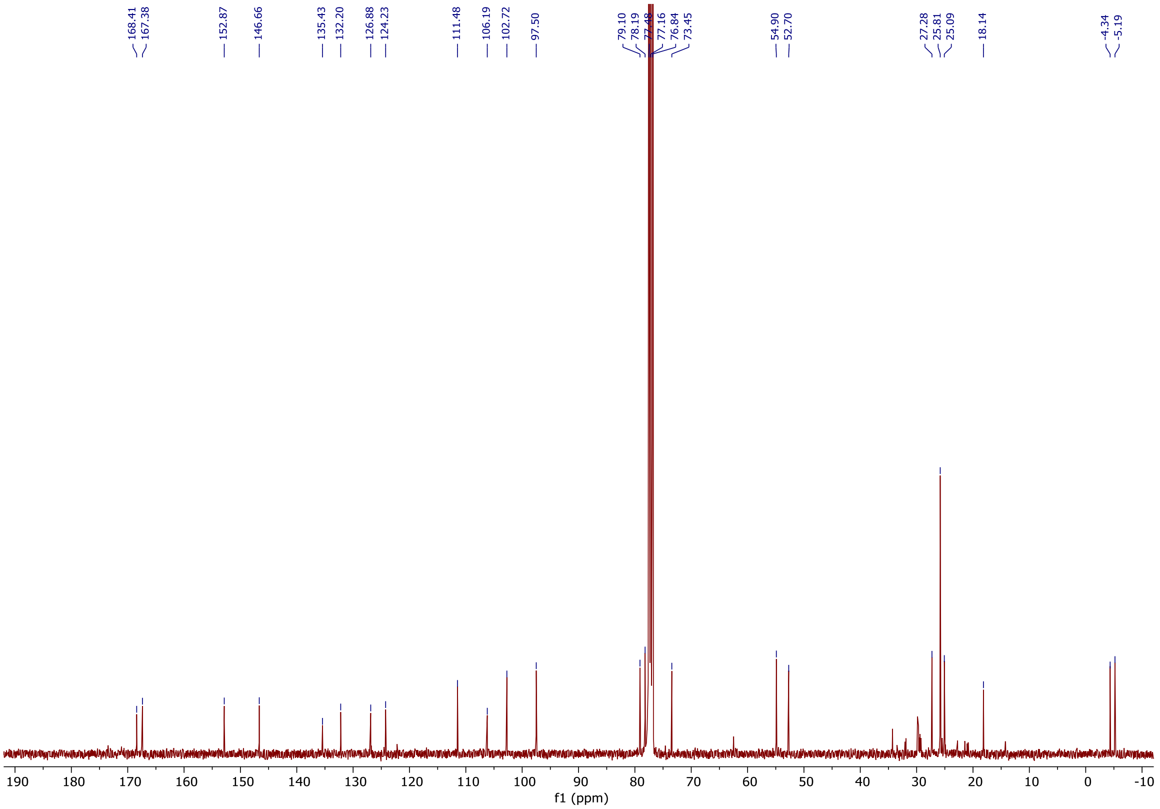

168.41
167.38
152.87
146.66
135.43
132.20
126.88
124.23
111.48
106.19
102.72
97.50
79.10
78.19
77.16
76.84
73.45
54.90
52.70
27.28
25.81
25.09
18.14
-4.34
-5.19
f1 (ppm)

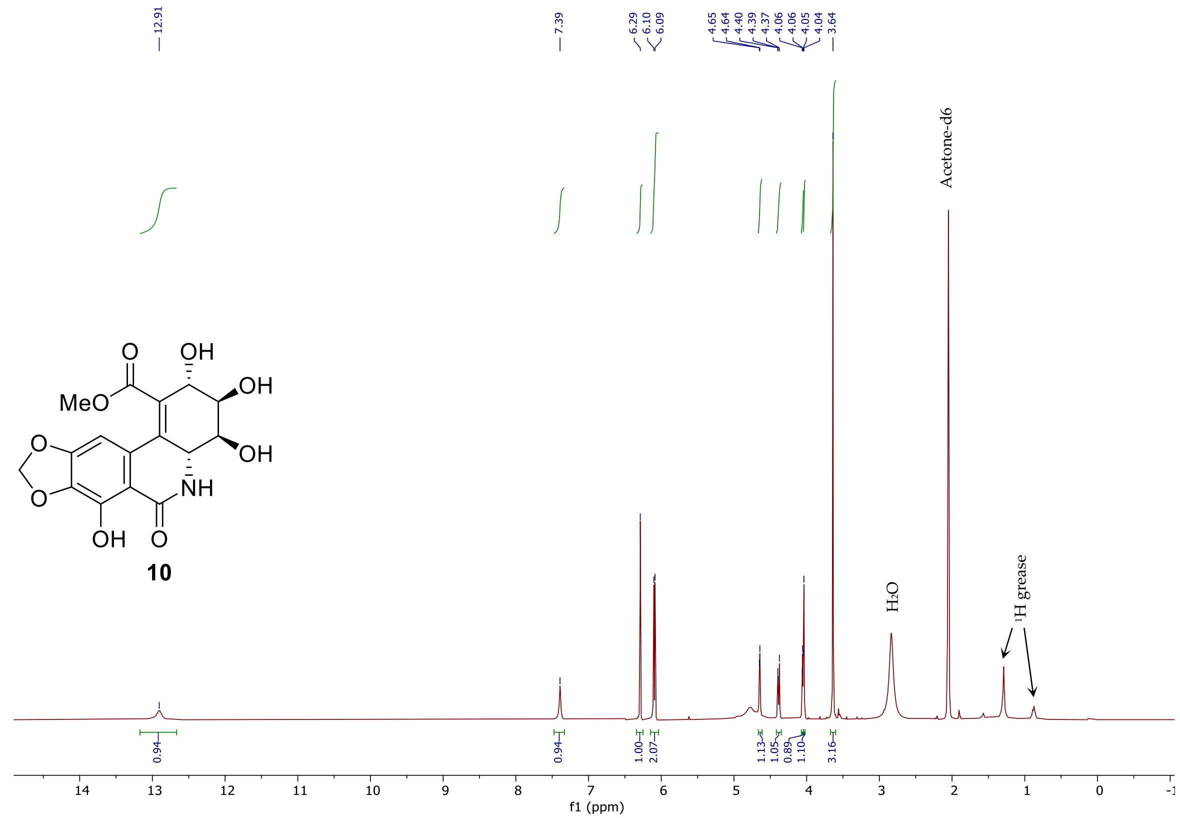

12.91
7.39
6.29
6.10
6.09
4.65
4.64
4.40
4.39
4.37
4.06
4.06
4.05
4.04
3.64
Acetone-d6
H2O
1H grease
0.94
0.94
1.00
2.07
1.13
1.05
0.89
1.10
3.16
14
13
12
11
10
9
8
7
6
5
4
3
2
1
0
f1 (ppm)
O
OH
MeO
OH
OH
O
O
NH
OH
O

10

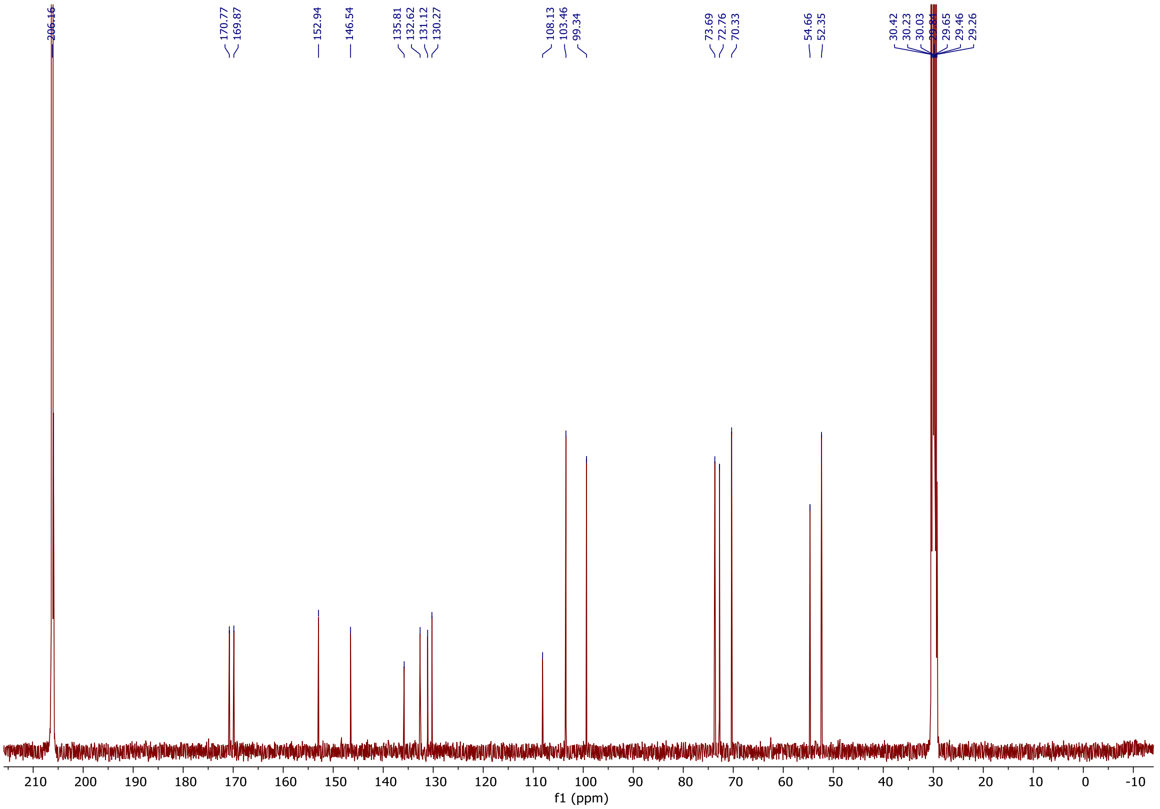

206.16
170.77
169.87
152.94
146.54
135.81
132.62
131.12
130.27
108.13
103.46
99.34
73.69
72.76
70.33
54.66
52.35
30.42
30.23
30.03
29.65
29.46
29.26
210
200
190
180
170
160
150
140
130
120
110
100
90
80
70
60
50
40
30
20
10
0
-10
f1 (ppm)
